# Periodontal status and oral health-related quality of life among rural population in Patna, India

**DOI:** 10.6026/973206300223562

**Published:** 2026-06-30

**Authors:** Soni Soni, Ravishankar Kumar, Garima Mangal, Nirmala Kumari

**Affiliations:** 1 Department of Public Health Dentistry, Buddha Institute of Dental Sciences, Patna, Bihar, India; 2 Department of Anatomy, Darbhanga Medical College and Hospital, Darbhanga, Bihar, India

**Keywords:** Community Periodontal Index, Gingival bleeding, Oral health-related quality of life, Periodontal disease, Rural population

## Abstract

Periodontal disease is highly prevalent in rural India, yet data linking clinical periodontal status with oral health-related quality of life (OHRQoL) in these communities remain scarce. Hence, a descriptive cross-sectional study was conducted among 550 rural adults in Patna using the WHO Oral Health Assessment Form (2013), modified CPI and OHIP-14 questionnaire from September 2020 to February 2021. Gingival bleeding was found in 48.73%, periodontal pockets (4-5 mm) in 60.36% and loss of attachment predominantly in the 0-3 mm range, with higher severity in females and older age groups. Higher OHIP-14 scores were significantly associated with gingival bleeding, periodontal pockets and loss of attachment (P < 0.001), confirming that worsening periodontal status adversely affects OHRQoL. This study provides novel region-specific evidence from rural Bihar by integrating clinical periodontal indicators with subjective OHIP-14 outcomes, supporting targeted oral health promotion and integration of dental care into primary healthcare for underserved populations.

## Background:

Periodontal diseases constitute a major public health concern worldwide, affecting a significant proportion of the adult population and contributing substantially to the global burden of oral diseases [1]. These conditions, primarily characterized by inflammation of the supporting structures of the teeth, range from mild gingivitis to severe periodontitis, which can ultimately result in tooth loss if left untreated [2]. The etiology of periodontal disease is multifactorial, involving the interaction of microbial plaque with host immune responses, along with various modifying factors such as age, socio-economic status, oral hygiene practices and deleterious habits like tobacco use [3]. In recent years, increasing attention has been directed toward understanding not only the clinical aspects of periodontal diseases but also their broader impact on an individual's overall well-being [4]. Oral health is an integral component of general health and significantly influences quality of life. Oral Health-Related Quality of Life (OHRQoL) is a multidimensional concept that reflects the impact of oral health on daily functioning, psychological well-being and social interactions [5]. Poor periodontal health can lead to symptoms such as bleeding gums, pain, halitosis and tooth mobility, which may adversely affect an individual's ability to eat, speak and engage confidently in social settings. Consequently, the assessment of periodontal status in relation to OHRQoL has become an important area of research, as it provides a more comprehensive understanding of the burden of oral diseases beyond clinical indicators alone [6]. Rural populations, particularly in developing countries like India, are often disproportionately affected by poor oral health due to limited access to dental care services, lack of awareness, lower socio- economic status and traditional oral hygiene practices. In such settings, preventive and curative dental services are frequently underutilized and oral diseases may remain untreated for prolonged periods [7]. Additionally, cultural beliefs, educational disparities and financial constraints further contribute to neglect of oral health, thereby exacerbating the severity of periodontal conditions. Despite the high prevalence of these issues, there remains a paucity of data focusing on the relationship between periodontal health and quality of life among rural communities [8]. Understanding this relationship is essential for developing targeted public health strategies and interventions aimed at improving oral health outcomes. By evaluating both clinical periodontal parameters and patient-reported outcomes, researchers and policy makers can gain valuable insights into the real-life impact of oral diseases on affected populations [9]. Such information is crucial for designing effective oral health promotion programs, enhancing accessibility to dental care and integrating oral health into primary healthcare systems [10]. Therefore, it is of interest to assess periodontal status and its influence on oral health related quality of life among rural adult populations.

## Methodology:

The survey was systematically organized following prior permission from the concerned authorities and ethical clearance was obtained from the Institutional Ethical Review Board after submission of the study protocol. Before the commencement of data collection, prescheduling of examination dates and times was carried out to ensure smooth coordination and subjects visiting on the scheduled days were included in the study. A pilot survey was conducted to evaluate the feasibility of the study, assess the clarity, validity and applicability of the questionnaire and standardize the examination procedure for recording periodontal status. Based on the findings of the pilot study, necessary modifications were made to the questionnaire and the required number of forms was finalized. The main survey was conducted over a period of six months from September 2020 to February 2021 using a descriptive cross-sectional study design among the rural population of Patna. The study population comprised 550 subjects aged 18 years and above residing in rural areas of Patna. Data were collected using a simple random sampling technique from selected villages. The sample size was calculated using G*Power software, considering a prevalence of periodontitis of 53% among females and 47% among males as observed in the pilot study; the calculated sample size was 532, which was increased to 550 to account for non-respondents. Inclusion criteria consisted of individuals aged 18 years and above who provided informed consent, while exclusion criteria included medically compromised individuals, those undergoing orthodontic treatment, patients with neurological or cognitive impairments, complete denture wearers and individuals with painful dental conditions. The study duration was six months. All clinical examinations were performed by a single calibrated investigator to ensure consistency. Prior to the study, the examiner underwent training and calibration under expert supervision to minimize intra-examiner variability. Calibration was performed on a small group representing a wide range of oral conditions and reliability was assessed by re-examining 10-12 subjects on different occasions. The kappa coefficient values for periodontal status and loss of attachment were 0.90 and 0.91, respectively, indicating a high degree of reliability. Subjects fulfilling the inclusion criteria were enrolled after obtaining written informed consent. A structured questionnaire was administered, followed by a detailed oral examination using the WHO Oral Health Assessment Form for Adults (2013). Oral health-related quality of life was assessed using the OHIP-14 questionnaire, which evaluates seven dimensions: functional limitation, physical pain, psychological discomfort, physical disability, psychological disability, social disability and handicap. The questionnaire employed a 5- point Likert scale ranging from "never" (score 0) to "very often" (score 4), with total scores ranging from 0 to 56, where higher scores indicated poorer oral health-related quality of life. The questionnaire was translated into Hindi using a standardized back-translation method and for illiterate participants, the examiner administered it verbally. Socioeconomic status was assessed using the Kuppuswamy Socioeconomic Status Scale (2020), which considers education, occupation and monthly family income to classify participants into five socioeconomic classes. Clinical examination was conducted using standard diagnostic instruments including mouth mirror, explorer, CPI probe and other sterilized armamentarium. Type III clinical examination as recommended by the American Dental Association was followed. Periodontal status was assessed using the modified Community Periodontal Index (CPI), recording gingival bleeding and periodontal pocket depth using a WHO CPI probe. Loss of attachment was evaluated sextant-wise using standardized criteria and coding. Strict sterilization protocols were followed, with instruments autoclaved and transported in sterile containers. Cold sterilization using chemical disinfectants such as glutaraldehyde-based solutions was also employed, with instruments immersed for at least 15 minutes. Additionally, comprehensive COVID-19 infection control protocols as per the Ministry of Health guidelines were implemented throughout the study. These included maintaining social distancing, limiting the number of participants in waiting areas, temperature screening, use of hand sanitizers, pre- procedural mouth rinsing with diluted povidone-iodine and use of full personal protective equipment (PPE) by the examiner and recorder. All biomedical waste was disposed of following standard protocols. Overall, the methodology ensured a systematic, standardized and ethically compliant approach for assessing periodontal status and its impact on oral health-related quality of life among the rural population.

## Results:

The study population consisted of 550 participants, with the majority belonging to the 31-40 years age group (214; 38.90%), followed by 21-30 years (189; 34.36%), 41-50 years (82; 14.90%) and >50 years (65; 11.82%). Females (287; 52.19%) marginally outnumbered males (263; 47.81%). The mean age among males was 34.98 ± 9.60 years, while females demonstrated a slightly higher mean age of 36.00 ± 10.20 years. Educational attainment was generally low, with most participants educated up to primary (161; 29.27%) and middle school levels (129; 23.45%), whereas only 36 (6.55%) belonged to the professional category. Occupationally, plant and machine operators constituted the largest group (164; 29.82%), followed by elementary workers (116; 21.09%). Socioeconomic assessment revealed that a majority belonged to the upper lower socioeconomic class (321; 58.36%), while 134 (24.36%) and 95 (17.27%) belonged to the upper middle and lower middle classes, respectively. Most participants were married (469; 85.27%) (Table 1). Assessment of oral hygiene practices revealed that toothbrushes were the most commonly used cleaning aid among 450 (81.82%) participants, whereas 66 (12.00%) relied on indigenous methods and 34 (6.18%) used fingers for cleaning teeth. Toothpaste was the predominant cleaning material used by 437 (79.45%) ndividuals, while tooth powder and charcoal were used by 63 (11.45%) and 36 (6.55%) participants, respectively. Brushing frequency was poor overall, with 516 (93.81%) brushing only once daily and merely 34 (6.18%) brushing twice daily. The combined brushing technique was most frequently practiced (361; 65.64%), followed by horizontal brushing (160; 29.09%), while vertical (14; 2.55%) and circular techniques (15; 2.73%) were uncommon. None of the participants reported using adjunctive oral hygiene aids such as dental floss, interdental brushes or mouthwash. Dental service utilization was notably low, with the majority reporting no previous dental visit. Among those who had visited a dentist, the commonest reasons included fillings (67; 12.18%), toothache (36; 6.55%), routine dental check-ups (32; 5.82%) and orthodontic alignment (24; 4.36%). Systemic diseases were reported among 60 (10.90%) participants, and most cases had duration of ≤1 year (77; 14.00%) (Table 2). Evaluation of adverse habits demonstrated a considerable prevalence of smokeless tobacco consumption, particularly khaini (104; 18.90%), followed by gutkha (50; 9.09%), pan masala (40; 7.27%) and mawa (1; 0.18%). The frequency of tobacco use was commonly reported as 6-10 times/day among 123 (22.36%) participants and <5 times/day among 74 (13.45%). Habit duration was most frequently between >1-≤5 years (106; 19.27%), followed by >5-≤10 years (75; 13.64%). Tobacco placement was predominantly in the labial mucosa (99; 18.00%) and buccal mucosa (72; 13.09%). The majority retained tobacco products for 5-10 minutes (137; 24.90%), with usage intervals commonly reported every 4-5 hours (108; 19.64%). Smoking prevalence was comparatively lower, with cigarette smoking being most common (63; 11.45%), followed by cigar (32; 5.82%) and beedi smoking (22; 4.00%). However, smoking-related associations were statistically non-significant (P > 0.05). None of the study participants reported alcohol consumption or pregnancy. Dietary habits revealed that most participants consumed a mixed diet (421; 76.55%), whereas 126 (22.90%) were vegetarians (Table 3). Periodontal assessment revealed gingival bleeding in 268 (48.73%) participants, while 270 (49.09%) showed no gingival bleeding and 10 (1.81%) were excluded. Periodontal pocket depth evaluation demonstrated that 332 (60.36%) participants had pockets measuring 4-5 mm, whereas 216 (39.27%) showed no periodontal pockets. No participants exhibited pockets ≥6 mm. The mean gingival bleeding score was 182.66 ± 149.53, while the mean periodontal pocket score was 182.66 ± 168.49. Loss of attachment (LOA) assessment showed that the majority of participants exhibited LOA within the 0-3 mm range, followed by moderate LOA of 4-5 mm, whereas severe LOA of 6-8 mm was least prevalent. Tooth-wise distribution demonstrated that the highest proportions of LOA were consistently observed in the 0-3 mm category across index teeth 16/17, 11, 26/27, 36/37, 31 and 46/47. Age-wise analysis indicated that younger age groups, particularly 21-30 and 31-40 years, predominantly demonstrated LOA within the 0-3 mm range, while severity gradually increased in older age groups. Females exhibited a slightly higher prevalence of LOA compared to males (Table 4). Evaluation of oral health-related quality of life using OHIP-14 demonstrated that most participants perceived minimal negative impact of oral conditions on daily life, as the majority selected "never" across most questionnaire domains including pain, discomfort, functional limitation and psychological distress. Overall OHIP-14 score distribution showed that 425 (77.27%) participants had scores between 45-56, while 125 (22.73%) scored between 31-44, indicating statistically significant findings (P < 0.0001). Age-wise comparison revealed the highest mean OHIP-14 score among the 31-40 years age group (48.51 ± 6.11), closely followed by 21-30 years (48.46 ± 6.18), whereas lower mean scores were observed among 41-50 years (47.75 ± 6.45) and >50 years (47.80 ± 6.01), with statistically significant differences (P < 0.001). Participants with gingival bleeding demonstrated higher OHIP-14 scores (49.18 ± 5.82) compared to those without gingival bleeding (47.39 ± 6.35). Conversely, lower OHIP-14 scores were observed among participants with periodontal pockets (47.10 ± 6.72) compared to those without pockets (49.36 ± 5.49). LOA also demonstrated significant association with OHIP-14 scores, with the highest scores observed in LOA score 2 (48.26 ± 5.92), followed by score 0 (48.67 ± 5.77) and score 1 (47.84 ± 6.70) (P < 0.001), thereby confirming a statistically significant relationship between periodontal status and oral health-related quality of life (Table 5). Analysis of responses to individual OHIP-14 items demonstrated that the majority of participants selected "never" or "hardly ever" across most domains, indicating limited perceived impairment in oral health-related quality of life. Difficulties in pronunciation were rarely reported, with 417 participants selecting "never." Similarly, 402 participants reported never experiencing discomfort while eating, and 522 participants indicated that their diet was never unsatisfactory because of oral problems. Psychological and social impacts were also minimal, as most participants denied embarrassment, irritability, or inability to function due to oral conditions. Severe impacts such as being "very often" affected were absent across almost all domains. These findings collectively suggest that although periodontal disease manifestations were clinically prevalent, their perceived influence on daily functioning and psychosocial well-being remained comparatively low among the rural population studied (Table 6).

## Discussion:

The present study evaluated the periodontal status and its impact on oral health-related quality of life (OHRQoL) among a rural population in Patna, revealing a significant association between clinical periodontal parameters and perceived quality of life. The findings demonstrated a high prevalence of gingival bleeding (48.73%) and periodontal pockets (60.36%), along with predominantly mild to moderate loss of attachment, indicating a considerable burden of periodontal disease in the study population. Despite this, most participants reported minimal impact on OHRQoL, which may reflect adaptation, low awareness, or differing perceptions of oral health in rural settings. These findings are consistent with the study by Dsouza *et al.* (2023) [11], which reported a significant association between loss of attachment and OHIP-14 scores, confirming that worsening periodontal status negatively influences quality of life. Similarly, Ng and Leung (2006) [12] observed that individuals with higher clinical attachment loss experienced significantly poorer OHRQoL across multiple domains, including physical pain and psychological discomfort. The present study aligns with these observations, as higher OHIP-14 scores were associated with gingival bleeding and loss of attachment. In agreement with these results, Grover *et al.* (2016) [13] found a strong positive correlation between periodontal parameters such as plaque index, gingival index and probing depth with OHIP-14 scores, particularly among rural populations. Their study also highlighted that rural populations tend to exhibit poorer periodontal health and greater impact on quality of life, which partially contrasts with the current findings where perceived impact remained low despite disease presence. This d iscrepancy could be attributed to differences in awareness, cultural perceptions and expectations regarding oral health. Furthermore, the study by Shamim *et al.* (2022) [14] demonstrated that periodontal disease significantly affects both OHRQoL and psychological factors such as self-esteem, with probing depth emerging as a significant predictor of poorer quality of life. This supports the present findings where clinical indicators like gingival bleeding and attachment loss showed significant associations with OHIP-14 scores. In contrast, some studies have reported weaker or non-significant associations. For instance, the study by Irani *et al.* (2022) [15] suggested variability in the impact of periodontal disease on OHRQoL depending on systemic conditions such as diabetes, indicating that the relationship may be influenced by additional health factors. Similarly, Hijryana *et al.* (2021) [16] reported no strong statistical association between periodontal status and OHRQoL in an older population, possibly due to differences in age group, perception, or adaptation to chronic conditions. Another important observation from the present study is the low utilization of dental services and absence of adjunctive oral hygiene practices among participants. This is in line with previous literature indicating that rural populations often have limited access to dental care and lower awareness levels, which contribute to poor periodontal outcomes. Additionally, the high prevalence of tobacco use observed in this study further exacerbates periodontal disease severity and aligns with established evidence linking tobacco consumption with poor periodontal health. A recent systematic review also supports these findings, concluding that periodontitis is significantly associated with poorer OHRQoL and the severity of disease correlates with greater impact on daily life. This reinforces the importance of assessing both clinical and subjective parameters in oral health research [17]. Sen *et al.* (2025) [18] also reported that periodontal disease adversely affects oral health-related quality of life, particularly in relation to functional limitation, psychological discomfort and social disability domains. Their findings emphasized that increased periodontal severity was associated with deterioration in patient-reported outcomes, further reinforcing the present study observations that worsening periodontal parameters correspond with altered OHIP-14 scores.

## Conclusion:

We show a significant relationship between periodontal status and OHRQoL. However, the relatively low perceived impact despite substantial disease burden highlights the need for increased awareness, early intervention and improved accessibility to oral healthcare services in rural populations.

## Figures and Tables

**Table 1:** Socio-demographic characteristics of study population (n = 550)

**Variable**	**Category**	**n (%)**
Age Group (years)	21-30	189 (34.36)
	31-40	214 (38.90)
	41-50	82 (14.90)
	>50	65 (11.82)
Gender	Male	263 (47.81)
	Female	287 (52.19)
Mean Age (years)	Male	34.98 ± 9.60
	Female	36.00 ± 10.20
Education	Primary	161 (29.27)
	Middle School	129 (23.45)
	Professional	36 (6.55)
Occupation	Plant & Machine Operators	164 (29.82)
	Elementary Workers	116 (21.09)
Socioeconomic Status	Upper Lower Class	321 (58.36)
	Lower Middle Class	95 (17.27)
	Upper Middle Class	134 (24.36)
Marital Status	Married	469 (85.27)

**Table 2:** Oral hygiene practices, dental utilization and systemic health (n = 550)

**Variable**	**Category**	**n (%)**
Oral Hygiene Method	Toothbrush	450 (81.82)
	Indigenous Methods	66 (12.00)
	Finger	34 (6.18)
Cleaning Material Used	Toothpaste	437 (79.45)
	Tooth Powder	63 (11.45)
	Charcoal	36 (6.55)
Brushing Frequency	Once Daily	516 (93.81)
	Twice Daily	34 (6.18)
Brushing Technique	Combined	361 (65.64)
	Horizontal	160 (29.09)
	Vertical	14 (2.55)
	Circular	15 (2.73)
Adjunctive Oral Hygiene Aids	Floss / Interdental Brush /	0 (0.00)
	Mouthwash	
Dental Visit History	No Prior Visit	Majority
Reason for Dental Visit	Filling	67 (12.18)
	Toothache	36 (6.55)
	Routine Check-up	32 (5.82)
	Alignment	24 (4.36)
Systemic Disease Presence	Present	60 (10.90)
Duration of Systemic Disease	≤1 Year	77 (14.00)

**Table 3:** Adverse habits, smoking and dietary patterns (n = 550)

**Variable**	**Category**	**n (%)**
Smokeless Tobacco Use	Khaini	104 (18.90)
	Gutkha	50 (9.09)
	Pan Masala	40 (7.27)
	Mawa	1 (0.18)
Frequency of Use	<5 times/day	74 (13.45)
	6-10 times/day	123 (22.36)
Duration of Habit	>1-≤5 years	106 (19.27)
	>5-≤10 years	75 (13.64)
Site of Placement	Labial Mucosa	99 (18.00)
	Buccal Mucosa	72 (13.09)
Retention Time	5-10 minutes	137 (24.90)
Usage Interval	4-5 hours	108 (19.64)
Smoking Habits	Cigarette	63 (11.45)
	Cigar	32 (5.82)
	Beedi	22 (4.00)
Statistical Significance (Smoking)	Association	Not Significant (P > 0.05)
Alcohol Consumption	Present	0 (0.00)
Pregnancy Status	Present	0 (0.00)
Dietary Pattern	Mixed Diet	421 (76.55)
	Vegetarian	126 (22.90)

**Table 4:** Periodontal status and clinical findings (n = 550)

**Variable**	**Category**	**n (%) / Mean ± SD**
Gingival Bleeding	Present	268 (48.73)
	Absent	270 (49.09)
	Excluded	10 (1.81)
Periodontal Pocket Depth	No Pocket	216 (39.27)
	4-5 mm	332 (60.36)
	≥6 mm	0 (0.00)
Mean Scores	Gingival Bleeding	182.66 ± 149.53
	Periodontal Pocket	182.66 ± 168.49
Loss of Attachment (LOA)	0-3 mm	Predominant
	4-5 mm	Moderate
	6-8 mm	Least
Tooth-wise LOA Distribution	16/17, 11, 26/27, 36/37, 31, 46/47	Highest in 0-3 mm range
Age-wise Trend	21-30 &s 31-40 years	Higher proportion in 0-3 mm
	Older Age Groups	Increased severity
Gender-wise Trend	Females	Slightly higher LOA prevalence

**Table 5:** OHIP-14 scores and association with periodontal parameters (n = 550)

**Variable**	**Category**	**n (%) / Mean ± SD**	**P-value**
OHIP-14 Score Distribution	45-56	425 (77.27)	<0.0001
	31-44	125 (22.73)	
Age-wise Mean OHIP-14 Score	21-30 years	48.46 ± 6.18	<0.001
	31-40 years	48.51 ± 6.11	
	41-50 years	47.75 ± 6.45	
	>50 years	47.80 ± 6.01	
Gingival Bleeding vs OHIP-14	Score 1	49.18 ± 5.82	<0.001
	(Present)		
	Score 0	47.39 ± 6.35	
	(Absent)		
Periodontal Pocket vs OHIP-14	Score 1	47.10 ± 6.72	<0.001
	(Present)		
	Score 0 (Absent)	49.36 ± 5.49	
Loss of Attachment (LOA) vs OHIP-14	Score 0	48.67 ± 5.77	<0.001
	Score 1	47.84 ± 6.70	
	Score 2	48.26 ± 5.92	

**Table 6:** Distribution of subjects according to responses of items of OHIP-14

**Sl. No**	**Items**	**Responses**				
		Never 0	Hardly ever (1)	Occasionally (2)	Fairly often (3)	Very often (4)
1	Have you had trouble pronouncing any words because of problems with your teeth, mouth or dentures?	417	120	11	0	0
2	Have you felt that your sense of taste has worsened because of problems with Your teeth, mouth or dentures?	116	142	200	90	0
3	Have you had painful aching in your mouth?	87	278	156	27	0
4	Have you found it uncomfortable to eat any foods because of problems with your teeth, mouth or dentures?	402	144	1	1	0
5	Have you been self-conscious because of your teeth, mouth or dentures	260	178	109	1	0
6	Have you felt tense because of problems with your teeth, mouth or dentures?	277	199	70	2	0
7	Has your diet been unsatisfactory because of problems with your teeth, mouth or dentures?	522	24	1	1	0
8	Have you had to interrupt meals because of problems with your teeth, mouth or dentures?	511	32	5	0	0
9	Have you found it difficult to relax because of problems with your teeth, mouth or dentures?	511	32	5	0	0
10	Have you been a bit embarrassed because of problems with your teeth, mouth or Dentures?	278	198	71	1	0
11	Have you been a bit irritable with other people because of problems with your teeth, mouth or dentures?	282	194	142	1	0
12	Have you had difficulty doing your usual jobs because of problems with your teeth, mouth or dentures?	523	21	3	1	0
13	Have you felt that life in general was less satisfying because of problems with your teeth, mouth or dentures?	524	21	3	0	0
14	Have you been totally unable to function because of problems with your teeth, mouth or dentures?	278	201	69	0	0
